# *PLCG1* gene mutation in a child with autoimmune hemolytic anemia: a case report

**DOI:** 10.3389/fped.2026.1884859

**Published:** 2026-09-25

**Authors:** Sarah W. Alawadhi, Hessah W. Alsalem, Maysoun Al Rushood

**Affiliations:** 1 Department of Pediatrics, College of Medicine, Health Sciences Center, Kuwait University, Kuwait City, Kuwait; 2 Pediatric Allergy and Clinical Immunology Unit, Mubarak AL Kabir Hospital, Kuwait City, Kuwait

**Keywords:** AIHA, anemia, immunodeficiency, next-generation sequencing, *PLCG1*

## Abstract

Autoimmune hemolytic anemia (AIHA) is a highly heterogeneous disease characterized by immune-mediated destruction of red blood cells through cellular and humoral mechanisms, complement activation, cytokine release, and bone marrow compensation. Clinically, AIHA exhibits a broad spectrum of severity, ranging from mild or compensated anemia to life-threatening anemia. Traditionally, therapeutic approaches have relied on corticosteroids, immunosuppressive agents, and splenectomy as the mainstay treatment. Increasing evidence suggests that genetic factors, including variants in the *PLCG1* gene—which plays a critical role in immune signaling and regulation—may contribute to disease pathogenesis. Herein, we report a case of a 13-year-old boy diagnosed with AIHA at 11 months of age. The patient initially presented with pallor and jaundice associated with a febrile illness. The diagnostic workup revealed anemia, reticulocytosis, a peripheral blood smear consistent with AIHA, low serum-free haptoglobin levels, and a positive direct Coombs test result. Next-generation sequencing identified a novel heterozygous *PLCG1* missense variant, c.2617C > T p.(Arg873Trp), which has not been previously reported. This case expands the spectrum of *PLCG1*-associated immune dysregulation and suggests that this variant may contribute to the development of AIHA. This report provides a rationale for functional studies on the role of *PLCG1* in human disease and immune dysregulation.

## Introduction

Autoimmune hemolytic anemia (AIHA) is an acquired hemolytic disorder characterized by the production of autoantibodies that target red blood cells (RBCs), resulting in their premature destruction. Hemolysis predominantly occurs extravascularly within the spleen and liver, although intravascular hemolysis may be observed in rare cases. Although the majority of AIHA cases are idiopathic, the condition may also occur secondary to malignancies, autoimmune diseases, or exposure to certain medications. In addition, evidence supports an association between AIHA and both genetic and congenital diseases. Notably, genetic susceptibility has been linked to mutations in specific human leukocyte antigen (HLA-B8) and Bw6 loci(1). These variants may contribute to the pathogenesis of AIHA through several mechanisms, including alterations in RBC membranes and surface antigens, disruptions in cellular metabolism, and immune dysregulation, making RBCs more susceptible to immune attacks.

Genes implicated in AIHA include human immunoglobulin heavy-chain variable region genes (*IGHV4-34* and *IGHV3*), an immunoglobulin kappa variable region gene (*IGKV3-20*), cytotoxic T-lymphocyte-associated protein 4 (*CTLA4*), and lymphotoxin-alpha (*LTA*), as well as more recently identified genes such as lysine methyltransferase 2D (*KMT2D*) and caspase recruitment domain family member 11 (*CARD11*) (1, 2). Among these genes, *CTLA4* and *PLCG1* are involved in immune tolerance and regulation, respectively. In this case report, we discuss the *PLCG1* gene, which encodes phospholipase C gamma 1, a signaling enzyme that is ubiquitously expressed in most cell and tissue types. It is highly expressed in neural tissue, implicating it in neurological development. Notably, dysregulation or aberrant expression of *PLCG1* has been associated with several neurological disorders, including epilepsy, movement and behavioral disorders, and neurodegenerative diseases (3). In addition to its role in the nervous system, *PLCG1* participates in angiogenesis, integrin-mediated cell motility, and apoptosis (4). Mutations in the *PLCG1* gene have been reported in association with a wide range of pathological conditions, including immune dysregulation, myelodysplastic syndrome (MDS), malignancies, infertility, cardiovascular diseases, and metabolic syndrome (3–8). These mutations have also been described in multiple case reports with variable manifestations, as presented in Tables 1A,B.

**Table 1:** A summary of previously reported cases with *PLCG1* mutations, illustrating the variability in clinical features, genetic variants, inheritance patterns, and investigations, compared to the present case.

A. Clinical features of patients with *PLCG1* mutation						
Patient characteristics	Patient 1 (9)	Patient 2 (9)	Patient 3 (9)	Patient 4 (9)	Patient 5 (9)	Patient 6 (9)
*PLCG1* variant	Missense (c.1789C > T p.(Leu597Phe)	Missense (c.1789C > T p.(Leu597Phe)	Missense (c.1789C > T p.(Leu597 Phe)	c.3056A > G, p.(Asp101 9Gly)	c.3494A > G, p.(Asp1165 Gly)	c.1139A > G, p.(His380Arg)
Inheritance pattern	Inherited	Inherited	Inherited	*de novo*, Sanger confirmed	*de novo*, Sanger confirmed	*de novo*, Sanger confirmed
Gender	Female	Female	Male	Male	Male	Female
Age at evaluation	11 years	42 years	6 years	18 years	9 years	14 years
Age of onset	Congenital	6 years	Childhood	Hearing loss since birth; other congenital anomalies recognized in infancy	Congenital	Congenital microphthalmia/optic atrophy; episodic steroid-responsive inflammatory encephalomyelitis/optic neuritis from 11 years
Presentation at evaluation	Bilateral congenital and progressive sensorineural hearing loss, mild motor delay, and unilateral posterior embryotoxon. A history of joint inflammation, tarsal synovitis, recurrent respiratory and lung infections, and inflammatory lymphadenopathy	Ventricular septal defect at birth. The patient had a severe immunologic history starting at the age of 6 years, with immune thrombocytopenic purpura, polyarthritis, autoimmune pulmonary fibrosis, and pneumococcal sepsis. The patient also presented with a congenital ventricular septal defect and high myopia since adolescence. She experienced sudden bilateral hearing loss during corticosteroid therapy, and had moderate bilateral sensorineural hearing loss by the age of 42 years	History of recurrent upper respiratory and lung infections. Hearing was normal	Presented with multiple joint contractures, stiffness, and difficulty with gait. He was also diagnosed with pyloric stenosis, congenital mild hearing loss, bilateral posterior embryotoxon with Axenfeld anomaly, and a ventricular septal defect. He had gross motor delay, partly related to multiple joint contractures since infancy	The patient had a history of intrauterine growth retardation. He had a mild-to-moderate sensorineural deafness since birth. He also presented with a small subaortic ventricular septal defect and a wide atrial septal defect with a left-to-right shunt. He had recurrent pulmonary infections in the first year of life and absolute T lymphocytopenia with normal absolute B lymphocytes. He presented with some facial dysmorphisms such as left eyelid ptosis, a downturned mouth, a long and flat philtrum, anteverted nostrils, prognathism, and evident veins of the scalp	The patient had left congenital microphthalmia and right-sided optic nerve hypoplasia. At the age of 11 years, she presented with a slowly progressive relapsing inflammatory encephalomyelitis with optic neuritis and a leukoencephalopathy that was rapidly responsive to corticosteroid treatment
Investigations	Normal hemato-immunologic tests.	Chronic thrombocytopenia and IgA and IgG2 deficiency.	Mild IgA and IgG2 deficiency.	- Brain MRI revealed stable mild diffuse cerebral and cerebellar volume loss and stable multifocal gliosis within the supratentorial white matter. - Spine MRI was consistent with mild caudal regression syndrome.		- Brain MRI showed progressive strikingly symmetric changes, consisting of primarily white matter swelling, persistent diffuse T2 FLAIR, and confluent hyperintensities within the frontoparietal lobes bilaterally. - Apart from a slightly reduced NK cell CD56 level), there was no obvious immunodeficiency identified.

B. Clinical features of patients with PLCG1 mutation			
Patient characteristics	Patient 7 (3)	Patient 8 (9)	Patient 9 (our patient)
Diagnosis	Immune dysregulation disease	CD4−CD8+ ATLL	AIHA
*PLCG1* variants	Gain of function, c.3062C > T, p.S1021F	Missense (c.1021G > A; p.G341R)	Missense variant c.2617C > T p.(ARG873Trp)
Gender	Female	Male	Male
Age at evaluation	7 years	76 years	11 months
Presentation at evaluation	Recurrent skin ecchymosis on the bilateral lower limbs gradually expanded to the abdomen and right upper limb, causing epistaxis and gingival bleeding. Cervical and inguinal lymphadenopathy was also noted	Unintentional weight loss and night sweats over 3 months and generalized lymphadenopathy	Progressive pallor and jaundice following a febrile illness
Investigations	Blood profile: low levels of hemoglobin, thrombocytopenia. Low complement 3 and 4. ESR: elevated. Immunological studies: positive antinuclear antibodies, anti-SSA antibodies, antinucleosome antibodies, and antiplatelet glycoprotein IIb/IIIa antibodies. Coombs test result: positive. Quantification of lymphocyte subsets: normal percentage of T and B lymphocytes, and a reduced proportion of natural killer cells. Bone marrow biopsy: marrow hypercellularity with active myelopoiesis and erythropoiesis but impaired platelet production. Thalassemia genetic testing revealed traits for α-thalassemia and β-thalassemia	Blood profile: anemia, mild thrombocytopenia, and mildly elevated LDH levels. CT scan: diffuse hilar and inguinal lymphadenopathy. Fine needle aspiration cytology: Paucicellular with some large atypical cells concerning for lymphoma. Flow cytometry revealed a population of atypical T cells that were positive for CD2, CD7 (dim), and CD8, and negative for surface CD3, CD5, and CD4. Immunohistochemical staining: atypical lymphocytes were positive for CD3, CD8, and CD25; variably positive for CD2, CD7, and TIA-1; and negative for CD4, CD5, CD20, CD34, TdT, EBER, and CD30. Ki-67 demonstrated a high proliferation index (>90%). HTLV-1 serology: reactive	Blood profile: low hemoglobin levels and elevated reticulocyte count. Free haptoglobin: low. Peripheral blood smears showed nucleated RBCs, anisopoikilocytosis, spherocytes, and polychromatic RBCs. Direct Coombs test result: positive. LDH: high

EBER, Epstein-Barr Virus-Encoded RNA.

*PLCG1* is a protein-coding gene involved in the regulation of intracellular signaling cascades and the generation of second messenger molecules essential for both the innate and adaptive immune systems. It functions as a regulatory enzyme in multiple pathways, including the nuclear factor-κappa B (NF-κB), extracellular signal-regulated kinase (ERK), mitogen-activated protein kinase (MAPK), and nuclear factor of activated T-cell (NFAT) signaling pathways (5). PLCG1 catalyzes the cleavage of phosphatidylinositol 4,5-bisphosphate into inositol 1,4,5-trisphosphate (IP3) and diacylglycerol (DAG). IP3 binds to its receptor (IP3R) and triggers a flux of calcium ions (Ca^2+^), which are second messengers that activate NFAT. Simultaneously, DAG mediates the activation of protein kinase C (PKC), driving downstream signaling through pathways such as NF-κB, ERK, and MAPK (3, 5).

The transcription factor NF-κB regulates multiple aspects of innate and adaptive immune responses and functions as a proinflammatory mediator (10). NF-κB plays a crucial role in establishing immune tolerance, encompassing both central tolerance and peripheral functions of regulatory T cells (Tregs). Thus, defective or deregulated activation of NF-κB can contribute to autoimmunity and inflammation. Signaling via ERK1/2, the terminal component of the MAPK phosphorylation cascade, plays a central role in fundamental cellular behaviors such as survival, proliferation, differentiation, and migration and critically shapes the inflammatory response and immune cell fate. ERK1/2 has been associated with several immune-mediated diseases characterized by chronic inflammation (11). Similar to the NF-κB pathway, NFAT has been shown to control Treg function (12).

In this case report, we describe a second case of immune dysregulation associated with a *PLCG1* gene mutation and compare our patient's clinical presentation with previously reported cases carrying *PLCG1* mutations.

## Case description

We present the clinical case of a 13-year-old Egyptian boy with a history of AIHA diagnosed at 11 months of age who was found to carry a *PLCG1* gene mutation. The patient was born to non-consanguineous parents through an uncomplicated full-term spontaneous vaginal delivery following an uneventful pregnancy and perinatal and postnatal periods. He achieved normal developmental milestones and demonstrated normal academic performance.

The family had a history of autoimmune disease. Although both parents were healthy, the patient's brother had a history of childhood atopy that resolved with age. The maternal grandmother and maternal great-grandmother had type 1 and type 2 diabetes mellitus, respectively. The maternal grandfather died of stage IV gastric cancer. There was no family history of any hematological disorders.

The patient was initially admitted to the pediatric ward at 11 months of age, when he presented with progressive pallor and jaundice following a febrile illness. The diagnostic workup confirmed the diagnosis of AIHA. Upon admission, a complete blood count revealed a low hemoglobin level of 4.3 g/dL, an elevated platelet count of 506 × 10^9^/L, an elevated reticulocyte count of 32%, an elevated total bilirubin level of 38 μmol/L, and a low free haptoglobin level of 0.06 g/L. Peripheral blood smears revealed nucleated RBCs, anisopoikilocytosis, spherocytes, and polychromatic RBCs. Further investigations revealed a positive direct Coombs test (DCT) result and an elevated lactate dehydrogenase (LDH) level of 283 IU/L. The white blood cell count was elevated at 18.7 × 10^9^/L, with a neutrophil-predominant differential (absolute neutrophil count of 9.7 × 10^9^/L). Serum complement levels were low, with C3 and C4 levels of 0.747 and 0.107 g/L, respectively. A physical examination revealed pallor, jaundice, fever, and tachycardia (210 beats/min). There was no associated lymphadenopathy, and chest and abdominal examinations were unremarkable.

The patient relapsed and was readmitted at 1 year and 5 months of age. Although the patient usually presented with upper respiratory tract infections or otitis media, there was no history of other severe or significant infections. During these initial two admissions, the patient received multiple packed RBC transfusions, intravenous (IV) pulse methylprednisolone at 30 mg/kg, oral prednisolone at 1 mg/kg, mycophenolate mofetil, and oral and IV antibiotics. The patient responded well to treatment, with a favorable hematological response and overall clinical improvement. An immunologist was consulted for the first time at 3 years of age to rule out underlying immunodeficiency or immunodysregulation as a cause of his refractory condition.

The patient experienced several severe relapses and remissions of AIHA following febrile illnesses, necessitating hospitalization, multiple blood transfusions, corticosteroid therapy, and eventually immunosuppressants. Relapses continued until 9 years of age, when treatment was escalated to IV rituximab, which was tolerated well. The patient received four courses of IV rituximab, with each course consisting of four weekly doses (16 mg/kg/dose) administered over 4 weeks. Maintenance treatment then focused on rituximab and mycophenolate mofetil, with the patient receiving his final course of mycophenolate mofetil at 9 years of age.

Since then, the patient has maintained normal hemoglobin levels and experienced multiple febrile illnesses that did not trigger a severe AIHA relapse or require hospitalization. The patient was subsequently permitted to resume his routine vaccination schedule, which had been paused early in life, except for the live-attenuated vaccines. One year later, both antidiphtheria and antipneumococcal capsular polysaccharide IgG titers were normal; however, antitetanus toxoid IgG titers remained low. Following booster administration, antitetanus toxoid IgG antibody levels increased, indicating adequate immunity. Since then, the patient has remained clinically stable with daily folic acid supplementation.

At 12 years of age, genetic testing through next-generation sequencing using the Illumina Exome 2.0 Plus assay identified a heterozygous missense variant, c.2617C > T p.(Arg873Trp), in the *PLCG1* gene (transcript NM_002660.2). The Arg873 residue is located within the split pleckstrin homology domain, which is a critical functional region of PLCG1. In this variant, arginine—a positively charged, basic, polar, hydrophilic amino acid—is replaced with tryptophan, an aromatic amino acid (13). Neither Sanger confirmation nor parental segregation testing was performed. This variant was absent from both the gnomAD genomes and gnomAD exome datasets despite good coverage (PM2). Moreover, the gnomAD missense *Z*-score for *PLCG1* was 3.24, exceeding the gene constraint threshold of 3.12 (PP2). *In silico* predictive tools yielded conflicting interpretations regarding pathogenicity, with a MetaRNN score of 0.418, which falls within the indeterminate range of 0.267–0.43 (BP4).

Follow-up laboratory evaluations at 13 years of age demonstrated normal hemoglobin levels, a normal reticulocyte count, and a peripheral blood smear showing normocytic and normochromic RBCs. At 12 years of age, serum haptoglobin levels and immunoglobulin assay results were normal, although the DCT remained positive. Lymphocyte immunophenotyping revealed normal B-cell and T-cell counts, with 21.04% CD19^+^ B cells, 53% CD3^+^ T cells, and 22.84% CD20^+^ B cells, alongside normal CD4^+^ and CD8^+^ subset counts and ratios. An immune evaluation revealed low serum immunoglobulin levels—including an IgG level of 4.26 g/L (reference range 5.4–16.1 g/L) and an IgM level of 0.371 g/L (reference range 0.5–1.8 g/L)—along with a reduction in class-switched memory B-cell numbers. The patient was diagnosed with possible evolving common variable immunodeficiency (CVID). The timeline figure (Figure 1) summarizes the clinical events of the patient.

**Figure 1:**
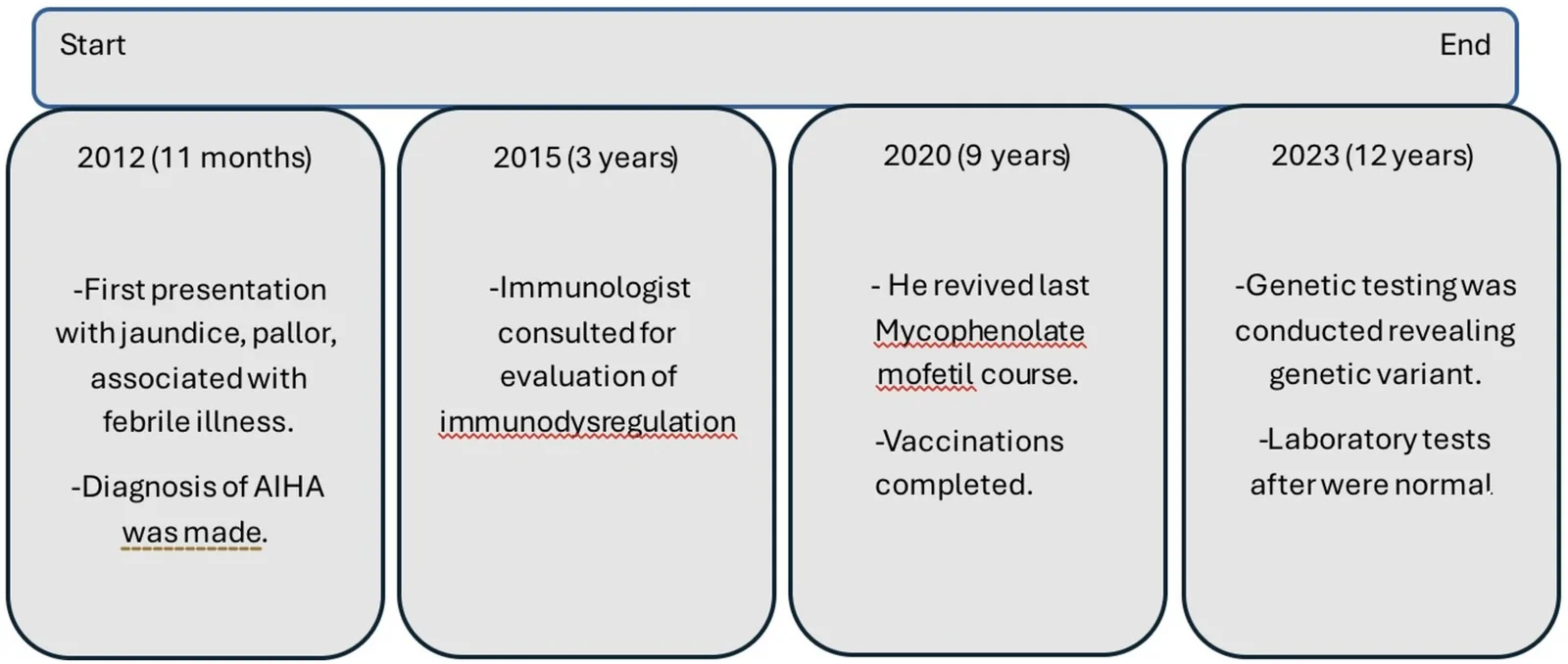
Timeline of the case events.

## Discussion

This case represents the first reported instance of a patient with a *PLCG1* mutation presenting with AIHA featuring a novel missense variant c.2617C > T p.(Arg873Trp). Most *PLCG1* variants reported to date are missense mutations, with gain-of-function variants specifically implicated in immune dysregulation. The clinical presentation and laboratory findings of the patient were consistent with a diagnosis of AIHA. However, the refractory disease course prompted further immunological evaluation, which revealed reduced class-switched memory B cells, suggesting a possible evolving CVID. The novelty of the identified variant, together with the patient's longitudinal follow-up, which allowed for a detailed assessment of disease progression and treatment response, represents a major strength of this report. Furthermore, a comparison with previously reported *PLCG1*-associated disorders expands the current understanding of the phenotypic spectrum of *PLCG1*-related immune dysregulation. However, this study also has several limitations. Because this is a single case report, a causal relationship between the identified variant and AIHA could not be established. In addition, Sanger confirmation and family segregation analyses were not performed, limiting the ability to confirm the pathogenicity of the variant and its role in disease development. Therefore, additional studies and the identification of similar cases are required to validate the association between *PLCG1* variants and AIHA. Examining the underlying mechanisms of AIHA helps clarify how *PLCG1* dysfunction drives immune dysregulation observed in this patient.

Studies investigating the pathophysiological mechanisms of AIHA have reported reduced levels of interferon-gamma (IFN-γ), which normally suppresses the T-helper 2 (Th2) pathway. This reduction can lead to insufficient Th2 inhibition, thereby promoting the amplification of autoantibody-mediated autoimmune responses. In addition, cell-mediated immunity is upregulated in AIHA, as evidenced by the increased activity of cytotoxic CD8^+^ T lymphocytes, natural killer (NK) cells, and activated macrophages. These alterations result in the production of immunoglobulin G (IgG) autoantibodies, which lead to RBC destruction through antibody-dependent cellular cytotoxicity. Cells of the monocyte–macrophage system drive this process by phagocytosing coated RBCs. Furthermore, the ratios of lymphocyte subsets responsible for downregulating autoimmune responses, such as peripheral CD4^+^ Tregs, are reduced in patients with AIHA, contributing to heightened autoimmune activity (2).

In cases involving gain-of-function variants of the *PLCG1* gene, IP3 production and intracellular Ca^2+^ release are increased, and the phosphorylation levels of ERK, p65, and p38 are elevated. Single-cell transcriptomic and protein expression analyses have demonstrated that these variants induce an exacerbated inflammatory response in T cells and monocytes. Specifically, activating *PLCG1* variants enhances the NF-κB and type II IFN pathways in T cells while hyperactivating the NF-κB and type I IFN pathways in monocytes. Heterozygous mutations in *PLCG1*, located on chromosome 20q12, are associated with immune dysregulation, autoimmunity, and autoinflammation (IDAA). IDAA is an immunological disorder characterized by anemia and thrombocytopenia associated with circulating autoantibodies, a positive Coombs test result, and elevated levels of proinflammatory cytokines (5).

As noted previously, PLCG1 catalyzes the initiation of Ca^2+^ flux, which triggers NFAT activation, while DAG mediates PKC activation, driving downstream signaling through NF-κB, ERK, and MAPK, all of which play key roles in immune regulation. Thus, in this case, a missense variant affecting a functional region of the *PLCG1* gene is expected to disrupt immune system regulation, leading to downstream inflammation and immune dysregulation.

In addition to immune dysregulation, *PLCG1* mutations have been found to influence various hematologic and vascular malignancies. These include myelodysplastic syndromes, cutaneous T-cell lymphoma, angioimmunoblastic T-cell lymphoma, angiosarcoma (including Kaposi sarcoma), and hepatic and cardiac angiosarcomas (14, 15). The most prevalent mutation is the missense variant p.R707Q (arginine at position 707 is replaced by glutamine) within the highly conserved cSH2 autoinhibitory domain, resulting in constitutive activation of PLCG1 (14). *PLCG1* has also been implicated in MDS, where reduced PLCG1 expression is associated with disease pathogenesis. This deficiency predisposes patients to significant complications such as anemia, recurrent infections, and hemorrhagic manifestations, indicating that *PLCG1* is essential for normal erythropoiesis (6). Further supporting its oncogenic potential, *PLCG1* is the most frequently mutated gene in adult T-cell leukemia/lymphoma (ATLL), indicating that PLCG1 plays a major role in driving T-cell activation (8, 15).

The oncogenic effects of PLCG1 extend to a broad range of aggressive solid tumors, including carcinomas of the breast, lung, pancreas, stomach, prostate, and ovaries. Given the elevated expression of growth factor receptors, including the epidermal growth factor receptor, platelet-derived growth factor receptor, and vascular endothelial growth factor receptor, in many solid tumors, it is plausible that growth factor–induced hyperactivation of PLCG1 leads to uncontrolled cellular proliferation, a hallmark of tumorigenesis (3). In addition to its roles in oncogenesis and immune dysregulation, *PLCG1* has been associated with metabolic syndrome. Three genetic loci have been mapped in or near genes previously implicated in atherogenic dyslipidemia, vascular inflammation, type 1 diabetes, vascular dysfunction, and central adiposity (7).

## Conclusions

This report describes a patient with AIHA carrying a *PLCG1* missense variant, c.2617C > T p.(ARG873Trp). Given the critical role of *PLCG1* in immune signaling, this variant may contribute to immune dysregulation, autoimmunity, and immunodeficiency. As the role of the *PLCG1* gene in human health and disease remains incompletely understood, further research is needed to determine the pathogenic impact of this variant.

## Data Availability

The original contributions presented in the study are included in the article/supplementary material. Further inquiries can be directed to the corresponding author.
